# A Systematic Review and Meta-Analysis Evaluating the Surgical Outcomes of Progressive Tension Suturing Compared to Drains in Abdominoplasty Surgery

**DOI:** 10.1093/asj/sjae171

**Published:** 2024-07-30

**Authors:** Gautham Rao, Kian Daneshi, Alessandra Ceccaroni, Antonioenrico Gentile, Hafiz El-Shazali, Niamh Owens, Krishna Vyas, Ankur Khajuria

## Abstract

Closed suction drains are placed to prevent seroma formation after abdominoplasty, but evidence of their effectiveness is limited, and they may increase infection risk and patient discomfort. Previous meta-analyses comparing progressive tension suturing (PTS) to drainage (D) in abdominoplasty have been methodologically weak and small in sample size. In this study we aimed to conduct the first robust systematic review comparing PTS and D outcomes in abdominoplasty. The study was registered on PROSPERO (CRD42022346106). We searched MEDLINE, Embase, the Cochrane Central Register of Controlled Trials, Google Scholar, and Web of Science from September 19, 2022, to February 19, 2024. Data were pooled with a random effects Mantel–Haenszel model. Risk of bias was assessed with Cochrane's risk-of-bias tool and the ROBINS-I tool for randomized controlled trials and observational studies, respectively. The GRADE (Grading of Recommendations, Assessment, Development, and Evaluation) system evaluated methodological quality. PTS significantly reduced postoperative seroma rates (relative risk [RR] 0.34; 95% CI 0.15-0.76; *P* = .001) and reoperation rates (RR = 0.56; 95% CI 0.03-9.77; *P* = .05) compared to drains, with no significant differences in hematomas, infections, or dehiscence. The review included 24 studies with 750 patients, including 2 randomized controlled trials, and was found to be methodologically superior by AMSTAR 2 criteria. Subgroup analysis indicated that combining liposuction with PTS significantly reduced seromas (RR 0.18; 95%CI 0.00-7.39; *P* < .00001), infections (RR 0.16; 95% CI 0.03-0.86; *P* = .03), and dehiscence (RR 0.11; 95% CI 0.01-1.01; *P* = .05). This robust meta-analysis showed that PTS was more effective than drains in reducing seroma and reoperation rates, with no difference for hematomas or infections. Combining liposuction with PTS may be superior to placing drains. Larger, high-quality studies are needed to further assess the safety and efficacy of drainless abdominoplasty.

**Level of Evidence: 1:**

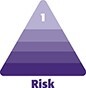

Abdominoplasty is the fourth most-performed aesthetic surgical procedure globally. Despite its widespread adoption, a preeminent challenge in abdominoplasty is the management of postoperative complications. However, the reported rate of complications associated with abdominoplasties has been widely variable at 4% to 80%.^1,2^ The dead space and wound tension created by extensive undermining and advancement of abdominal flaps are the main causes of local complications in abdominoplasty. The most encountered complications following abdominoplasty procedures include seromas, hematomas, wound healing problems, infection, contour irregularities, and pulmonary embolisms.^3,4^ Reported rates of seromas are 20% to 42%.^5-9^ These complications may lead to suboptimal results, significant morbidity, and increased duration of hospital stays.

Two popular techniques for reducing seroma formation and other complications postabdominoplasty are the placement of surgical drains and progressive tension suturing (PTS). Orthodox surgical drains function by removing excess fluid accumulation under the skin flap. Progressive tension sutures are thought to reduce the rate of seroma formation with sutures placed at intervals to remove dead space between the rectus abdominis fascia and the abdominal flap.^8^ This technique can be performed with or without the addition of suction drains.^2^ These are designed to remove any fluid that has accumulated between the 2 layers.^10^ This, however, has its own risks, including a greater likelihood of surgical site infections.^11^

Currently the decision to employ 1 technique over the other has been determined by the surgeon's preference, institutional norms, or specific patient characteristics, in a nonstandardized fashion. There is dissent over the superior method among previous studies. Nahas et al espoused PTS to decrease seroma formation rate, and further studies have followed this up by describing the efficacy of PTS in reducing seromas, reducing patient discomfort, and improving cosmetic results; on the other hand, other studies have reported on drains as a cost-effective, noninferior method for reducing seroma formation rate.^7,12,13^ Regarding efficacy in reducing postoperative complications, specifically comparing PTS with drains, Pollock and Pollock reported a reduced rate of seroma formations with PTS, whereas other studies have reported on noninferiority.^14^ However, 4 previously published systematic reviews (Li et al 2021, Ho et al 2020, Seretis et al 2017, and Jabbour et al 2016) comparing postoperative complications are of low or critically low quality as assessed by the AMSTAR 2 criteria (see Supplemental Table 1, located online at www.aestheticsurgeryjournal.com, which displays the quality of systematic reviews according to AMSTAR 2 criteria).^1,2,15,16^ This prohibits conclusions being drawn regarding the superior surgical method for reducing postoperative complications in abdominoplasty. Notably, there is an absence in the current literature of patient-reported outcomes (PRO) in establishing the relative superiority of PTS vs drains in abdominoplasty; this is an important avenue for future research efforts given that patient satisfaction is a central tenet of surgical success in aesthetic plastic surgery.

Our systematic review and meta-analysis, although addressing the methodological drawbacks of previous reviews with a robust prepublished methodology, aimed to assess the difference in the rates of seroma and hematoma formation as primary outcomes, as well as the difference in the rates of secondary outcomes, including wound dehiscence and infection, in abdominoplasty procedures with PTS vs drains. We also wished to address the impact of liposuction on discrepancies in postoperative outcomes.

## METHODS

The protocol for this study was registered and published a priori on PROSPERO (CRD42022346106).^17^ The AMSTAR 2 criteria were applied to assess the quality of previously published systematic reviews in relation to this review (see Supplemental Table 1, located online at www.aestheticsurgeryjournal.com).

### Search Strategy

The present systematic review and meta-analysis was compliant with PRISMA guidelines and was AMSTAR 2 compliant.^18,19^ Two authors independently searched MEDLINE (National Institutes of Health, Bethesda, MD), Embase (Elsevier, Amsterdam, the Netherlands), the Cochrane Central Register of Controlled Trials (Wiley, Hoboken, NJ), Google Scholar (Mountain View, CA), and Web of Science (Clarivate, London, UK) between September 19, 2022, and February 19, 2024, for studies published. The search strategy has been included in the Appendix (see Supplemental Table 2 located online at www.aestheticsurgeryjournal.com). No language restrictions were applied. For the included studies, reference lists were also screened for relevant articles.

Both randomized and observational studies on patients undergoing abdominoplasties with PTS or drains with 1 or more clinical outcomes of interest were included. Patient data collected included rates of hematoma and seroma formation, as well as reoperation, dehiscence, infections, cost, duration of hospital stay, and patient reported outcomes (PROs).

### Inclusion Criteria

- Studies involving patients ages over 18 years who had undergone abdominoplasty with progressive tension sutures, with or without drains.
- Clinical studies (randomized controlled trials [RCTs], prospective and retrospective cohort studies, and case series with more than 10 patients).

### Exclusion Criteria

- Review articles, clinical studies in nonhuman populations, simulation studies, case reports without outcome measures.
- Studies were screened for duplication; duplicates were excluded. Also, studies were screened for risk of bias. The reviewed Cochrane risk-of-bias tool was utilized to assess the quality of randomized controlled trials. Nonrandomized studies were evaluated with ROBINS-I. Studies deemed to be at serious or critical risk of bias were excluded.

**Figure 1.**
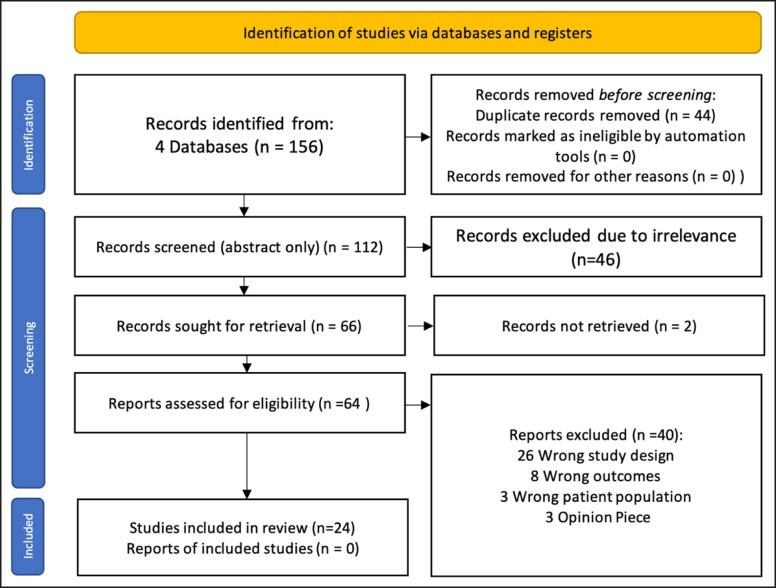
PRISMA flow diagram for this comprehensive meta-analysis and systematic review: the study selection and inclusion process.

We excluded studies if the inclusion criteria were not fulfilled or there were insufficient data to distinguish patient prognostics–based rates of hematoma and seroma formation. Furthermore, case series involving <20 patients, review articles, editorials, nonclinical studies, and conference abstracts were excluded. Additionally, studies in non-English languages without translation were excluded. The PRISMA flow diagram of selection of studies is depicted in Figure 1. The nature and characteristics of the studies included in this review are found in Supplemental Table 3, located online at www.aestheticsurgeryjournal.com. Preprints were deemed ineligible, owing to their preliminary nature.

Two independent authors screened the titles and abstracts of studies yielded in the search. Full texts of eligible papers were assessed for inclusion. Discrepancies in assessment were resolved by consultation with A.K.

### Data Analysis

Four authors independently extracted data from studies fulfilling the inclusion criteria. Study details (author, journal, date, country, study design, study period, and funding), total number of patients, and their postoperative outcomes categorized by abdominoplasty techniques were the principal included data points. Per AMSTAR 2, we planned to contact authors affiliated to papers with missing or incomplete data in studies of interest, endeavoring to obtain access to study-level anonymized data.^19^ The quality of the included studies was assessed with GRADE (Grading of Recommendations, Assessment, Development, and Evaluation) independently by 3 authors.^20^ We planned to modify this for case series, cohort studies, and cross-sectional studies. The cumulative risk scores were subsequently classified as low, moderate, or high risk of bias. A fourth author resolved any discrepancies in quality assessment.

Two authors independently assessed the quality of studies with ROBINS-I for observational studies and Cochrane risk of bias (RoB2) for randomized studies.^21,22^ Discrepancies in risk-of-bias assessments were addressed by consultation with A.K. Given the moderate to high expected heterogeneity associated with different clinical settings and variance among individual surgeons, and in line with the prespecified analysis methodology set out, random effects meta-analyses of pooled raw data were employed with the DerSimonian and Laird method according to the prespecified analysis plan, applied for each outcome with sufficient data to account for anticipated differences in study design over time. Outcomes from abdominoplasties with progressive tension sutures (PTS) were compared with outcomes from abdominoplasties with surgical drains. Adjusted effect estimates were combined where available, and, in the absence of adjustment for confounders, raw effect estimates were combined. For each outcome, the results were presented in forest plots as risk ratios (RRs), with corresponding 95% confidence intervals (CIs). I^2^ estimates of heterogeneity, representing the variability across studies, were classified as low (<30%), moderate (30%-60%), or high (>60%). Only good-quality studies were included for sensitivity analysis. Sensitivity analyses were preplanned to investigate how exclusive selection and analysis of low risk-of-bias studies and any RCTs yielded would affect the obtained results. The study protocol was prospectively registered with PROSPERO, no. CRD42022346106. Data were analyzed with RevMan (Review Manager) 5.4 (Cochrane Collaboration, 2020).

### Role of the Funding Source

No funding was received for the study. All authors had full access to all data and took responsibility for the decision to submit for publication.

## RESULTS

### Search and Included Studies

The literature search identified 156 studies. Following title and abstract screening, 66 studies were deemed eligible for inclusion following full-text screening. Hand search or snowballing of references of included full texts yielded no additional studies. Overall, the 24 studies selected included 4777 participants who underwent an abdominoplasty procedure, either with PTS, a surgical drain, or both. Of these participants, 2817 patients had PTS only, 1065 had surgical drains only, and 1346 patients had both. Several studies yielded in the search were single-arm studies (studies with only 1 experimental group), and these are summarized in Supplemental Table 3, located online at www.aestheticsurgeryjournal.com, for completeness, but our analysis focused on comparative study designs. Three of the included studies were RCTs, and the remainder were retrospective studies. Included studies were those comprising patients over 18 years old who had undergone abdominoplasty with progressive tension sutures, with or without drain usage. Studies describing other interventions were excluded (eg, Nagarkar et al investigated barbed PTS in deep inferior epigastric artery perforator [DIEP] flap donor-site closure).^23^

Clinical studies comprising RCTs and prospective and retrospective cohort studies were included.^9,10,24-58^ Excluded studies included review articles, clinical studies in nonhuman populations, simulation studies, and case reports without outcome measures. Publications that were a commentary on another piece of work, for example, TA Pollock 2020, and publications without the necessary outcome measures, for example, Fang et al, were excluded.^59,60^ The remaining studies were then screened for duplication and the duplicates excluded. Studies deemed to be at serious or critical risk of bias were also excluded. By AMSTAR 2 criteria the present review was deemed to be high quality and considerably more robust than previous reviews (Supplemental Table 1, located online at www.aestheticsurgeryjournal.com). Overall, the quality of individual studies was fair or good.

The PTS-only approach was applied in the largest group of studies (11 of 24, 45.8%), with the remainder opting for a surgical drain or combined approach. The PTS technique of choice varied between studies and included the interrupted suture PTS technique first described by Pollock and Pollock as well as continuous barbed PTS technique.^14^ The drain technique of choice also varied between studies and included closed suction drains, Hemovac drains (Zimmer Biomet, Warsaw, IN), and Jackson-Pratt drains (Cardinal Health, Dublin, OH).

### Clinical Outcomes

#### Postoperative Seroma

**Figure 2.**
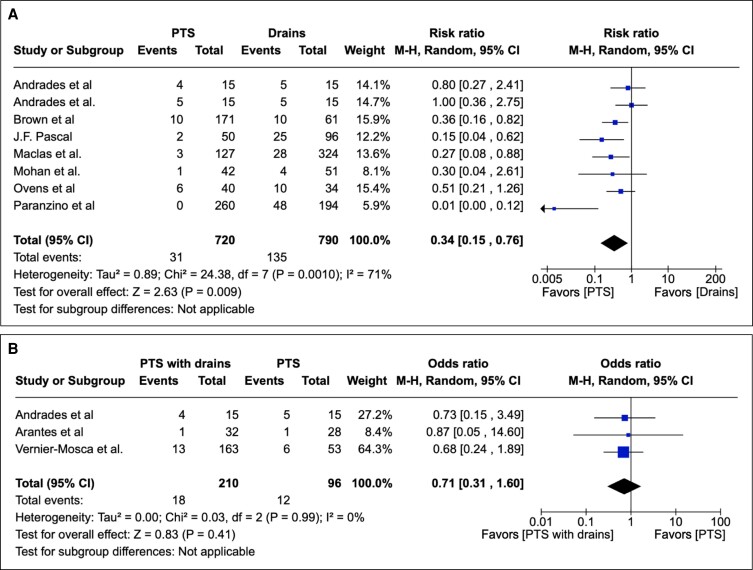
(A) Twenty-four studies pertaining to seromas containing patient groups treated with PTS or drains. Forest plot for 8 comparative studies with calculated M-H risk ratio for seromas in groups treated with abdominoplasties with PTS vs drains. (B) Twenty studies pertaining to seromas containing patient groups treated with PTS with drains or PTS. Forest plot for 3 comparative studies with calculated M-H risk ratio for seromas in groups treated with abdominoplasties with PTS with drains vs PTS. M-H, Mantel–Haenszel; PTS, progressive tension suturing.

Postoperative seroma was reported as an outcome in 24 studies as either an integer or percentage rate, facilitating calculation of the original integer. PTS, compared with conventional drains, was associated with a statistically significantly reduced seroma rate postoperatively in overall effect (RR 0.34; 95% CI 0.15-0.76; *P* = .0010). Assessing heterogeneity, Tau^2^ = 0.89; Chi^2^ = 24.38; df = 7 (*P* = .0010); and I^2^ = 46%. These results are depicted graphically in Figure 2A.

PTS with drains, compared with PTS, was not associated with a statistically significantly reduced seroma rate in overall effect (*P* = .71; 95% CI 0.31 to 1.60). For heterogeneity, Tau^2^ = 0.00; Chi^2^ = 0.03; df = 2 (*P* = .99); and I^2^ = 0%. These results are depicted graphically in Figure 2B.

#### Postoperative Hematoma

**Figure 3.**
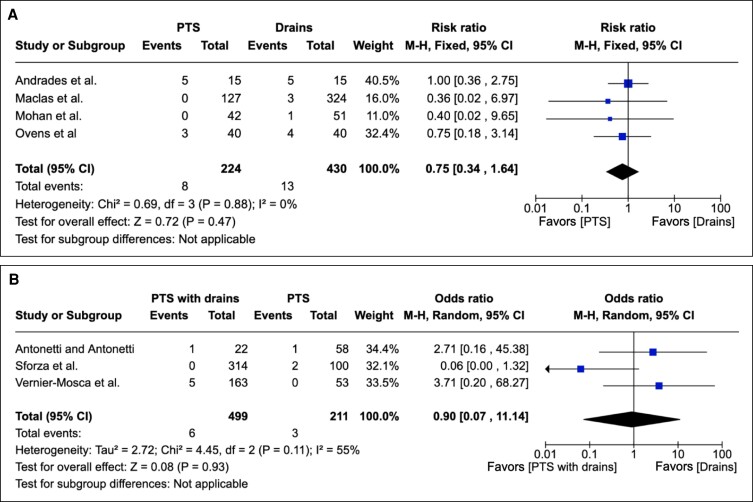
(A) Twenty-three studies pertaining to hematomas containing patient groups treated with PTS or drains. Forest plot for 4 comparative studies with calculated M-H risk ratio for hematomas with PTS vs drains. (B) Fifteen studies pertaining to hematomas containing patient groups treated with PTS with drains or PTS. Forest plot for 3 comparative studies with calculated M-H risk ratio for hematomas in groups treated with abdominoplasties with PTS with drains vs PTS. M-H, Mantel–Haenszel; PTS, progressive tension suturing.

Postoperative hematomas were reported as an outcome in 23 studies as either an integer or percentage rate. PTS when compared with drains was associated with no significant reduction of postoperative hematoma in overall effect (*P* = .88; RR = 0.75; 95% CI = 0.34 to 1.64). Heterogeneity reporting included Chi^2^ = 0.69; df = 2 (*P* = .47); I^2^ = 0%. These results are depicted graphically in Figure 3A.

PTS with drains, compared with PTS, was not associated with a statistically significantly reduced hematoma rate postoperatively in overall effect (*P* = .11; 95% CI 0.07 to 11.14). Regarding heterogeneity, Tau^2^ = 2.72; Chi^2^ = 4.45; df = 2 (*P* = .11); and I^2^ = 55%. These results are depicted graphically in Figure 3B.

#### Reoperation Rate

**Figure 4.**
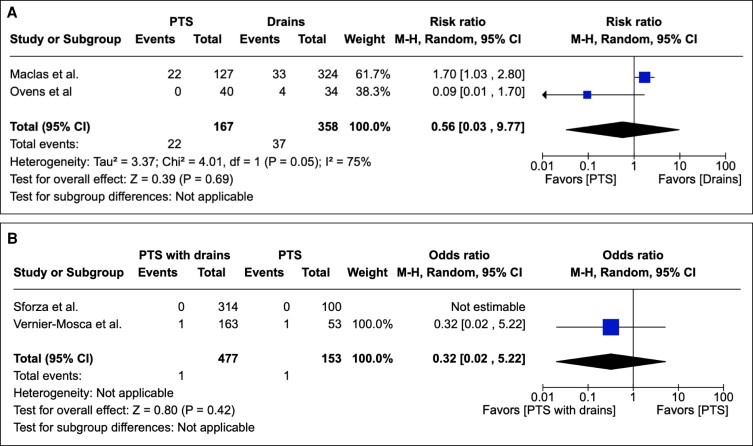
(A) Thirteen studies pertaining to reoperation rate containing patient groups treated with PTS or drains. Forest plot for 2 comparative studies with calculated M-H risk ratio for reoperation rate in groups treated with reoperation rates with PTS vs drains. (B) Nine studies pertaining to reoperation rate containing patient groups treated with PTS with drains or PTS. Forest plot for 2 comparative studies with calculated M-H risk ratio for reoperation rate in groups treated with abdominoplasties with PTS with drains vs PTS. M-H, Mantel–Haenszel; PTS, progressive tension suturing.

Reoperation rate was reported as an outcome in 13 studies. PTS when compared with conventional surgical drains was associated with a statistically significant decrease in reoperation rate (RR = 0.56; CI 0.03 to 9.77; *P* = .05; Tau^2^ = 3.37; Chi^2^ = 4.01; df = 1; I^2^ = 75%) derived from 2 comparative studies.^36,38^ Figure 4A depicts these results graphically.

PTS with drains, compared with PTS, was not associated with a statistically significantly reduced reoperation rate in overall effect (*P* = .42; 95% CI 0.34 to 5.22). Again, the high heterogeneity of results precluded any meaningful meta-analysis findings. These results are depicted graphically in Figure 4B.

#### Secondary Outcome: Infection

**Figure 5.**
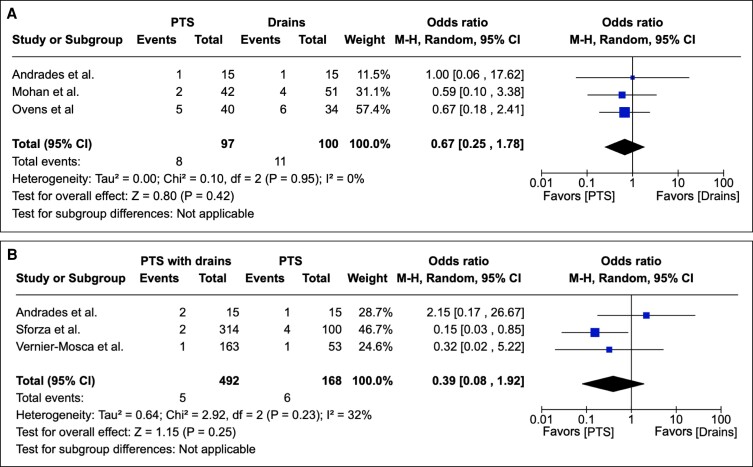
(A) Fourteen studies pertaining to infections containing patient groups treated with PTS or drains. Forest plot for 3 comparative studies with calculated M-H risk ratio for infections in groups treated with abdominoplasties with PTS vs drains. (B) Twelve studies pertaining to infections containing patient groups treated with PTS + drains or PTS. Forest plot for 3 comparative studies with calculated M-H risk ratio for infections in groups treated with abdominoplasties with PTS + drains vs PTS. M-H, Mantel–Haenszel; PTS, progressive tension suturing.

Infection was reported as an outcome in 14 studies as either an integer or percentage rate. PTS was associated with no statistically significant reduction in infection when compared with conventional surgical drains in overall effect (*P* = .95; 95% CI 0.25 to 1.78). Assessing heterogeneity, Tau^2^ = 0.00; Chi^2^ = 0.10; df = 2 (*P* = .95); I^2^ = 0%. Figure 5A depicts these results graphically.

Upon comparing PTS with drains with PTS, it was found that PTS with drains was not associated with a statistically significant reduction in infection postoperatively in overall effect (*P* = .23; 95% CI 0.08 to 1.92). Heterogeneity was assessed with the following values determined: Tau^2^ = 0.64; Chi^2^ = 2.92; df = 2 (*P* = .23); I^2^ = 32%. These results are depicted graphically in Figure 5B.

#### Secondary Outcome: Dehiscence

**Figure 6.**
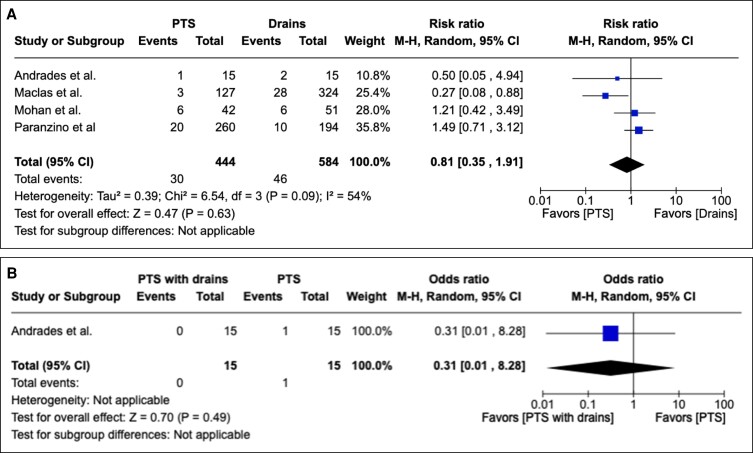
(A) Fourteen studies pertaining to dehiscence containing patient groups treated with PTS or drains. Forest plot for 4 comparative studies with calculated M-H risk ratio for dehiscence in groups treated with abdominoplasties with PTS + drains vs drains. (B) Ten studies pertaining to dehiscence containing patient groups treated with PTS or drains. Forest plot for 1 comparative study with calculated M-H risk ratio for dehiscence in groups treated with abdominoplasties with PTS with drains vs PTS. M-H, Mantel–Haenszel; PTS, progressive tension suturing.

Dehiscence was reported as an outcome in 14 studies as either an integer or percentage rate. PTS compared with the conventional method of surgical drainage did not yield a statistically significant reduction in dehiscence rate postoperatively in overall effect (*P* = .09; 95% CI 0.35 to 1.91). Heterogeneity assessment showed Tau^2^ = 0.39; Chi^2^ = 6.54; df = 3 (*P* = .09); and I^2^ = 54%. These data are depicted graphically in Figure 6A.

PTS with drains did not yield a statistically significant reduction in dehiscence rate postoperatively when compared to PTS alone in overall effect (*P* = .49; 95% CI 0.01 to 8.28). This information is depicted graphically in Figure 6B.

#### Impact of Liposuction on Progressive Suturing vs Drains Postoperative Outcomes

**Figure 7.**
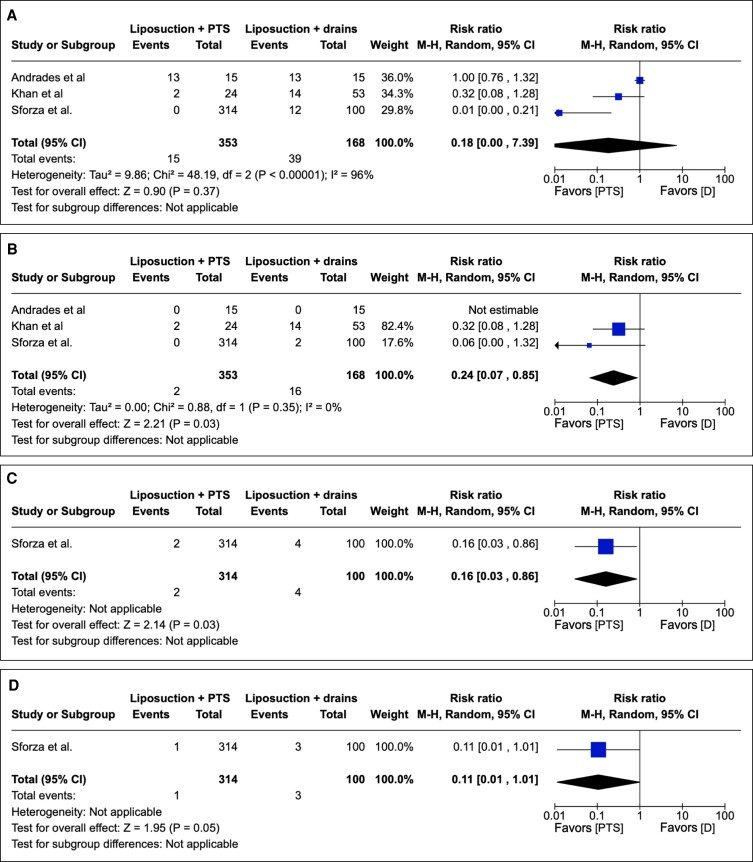
(A) Three studies pertaining to seromas containing patient groups treated with PTS or drains in 100% liposuction technique cohorts. Forest plot for 3 comparative studies with calculated M-H risk ratio for seromas in groups treated with abdominoplasties with PTS vs drains. (B) Three studies pertaining to hematomas containing patient groups treated with PTS or drains in 100% liposuction technique cohorts. Forest plot for 3 comparative studies with calculated M-H risk ratio for hematomas in groups treated with abdominoplasties with PTS vs drains. (C) One study pertaining to infections containing patient groups treated with PTS + drains or drains within the 100% liposuction subgroup. Forest plot for 1 comparative study with calculated M-H risk ratio for infections in groups treated with abdominoplasties with PTS vs drains. (D) One study pertaining to dehiscence containing patient groups treated with PTS + drains or drains within the 100% liposuction subgroup. Forest plot for 1 comparative study with calculated M-H risk ratio for dehiscence in groups treated with abdominoplasties with PTS vs drains. M-H, Mantel–Haenszel; PTS, progressive tension suturing.

To investigate the impact of liposuction on the postoperative outcomes with progressive tension suturing vs drains, we conducted further subgroup analysis of all postoperative outcomes in studies in which liposuction was performed on 100% of the cohort. In cohorts utilizing liposuction in 100% of cohorts, PTS, compared with conventional drains, was associated with a statistically significantly reduced seroma rate postoperatively in overall effect (RR 0.18; 95% CI 0.00-7.39; *P* < .00001). Assessing heterogeneity, Tau^2^ = 9.86; Chi^2^ = 48.19; df = 2 (*P* < .00001); and I^2^ = 96%. These results are depicted graphically in Figure 7A.

In the same 100% liposuction subgroup, PTS when compared with drains was associated with no significant reduction of postoperative hematoma in overall effect (*P* = .35; RR = 0.24; 95% CI = 0.07 to 0.85). Heterogeneity reporting included Chi^2^ = 0.88; df = 1 (*P* = .35); I^2^ = 0%. These results are depicted graphically in Figure 7B.

In the 100% liposuction subgroup, there were insufficient studies reporting data to calculate a risk ratio or draw conclusions, highlighting an area for future potential research. In this same subgroup, PTS was associated with a statistically significant reduction in infection when compared with conventional surgical drains in overall effect (RR = 0.16, *P* = .03; 95% CI 0.03 to 0.86). Figure 7C depicts these results graphically. Additionally, PTS compared with the conventional method of surgical drainage yielded a statistically significant reduction in dehiscence rate postoperatively in overall effect (RR = 0.11; *P* = .05; 95% CI 0.01 to 1.01). These data are depicted graphically in Figure 7D. Future studies should seek to disinter any potential discrepancy in postoperative outcomes between PTS + drains and PTS within the 100% liposuction subgroup, because presently such data are absent from the scientific corpus of knowledge.

## DISCUSSION

This is the first methodologically robust systematic review and meta-analysis comparing PTS vs drains in the literature. The strength of this study is its rigorous approach to comparing the best available evidence on the subject, pooling data from large cohorts and high-quality studies. Four previously published systematic reviews comparing postoperative complications were of low or critically low quality as assessed by the AMSTAR 2 criteria (see Supplemental Table 1, located online at www.aestheticsurgeryjournal.com, which displays the quality of systematic reviews according to AMSTAR 2 criteria), whereas our study was wholly AMSTAR 2 compliant and more rigorous. We have shown that rates of seroma are significantly lower with PTS than with drains, with no difference in the rate of hematoma, infection, or dehiscence between the 2 techniques. These findings are comparable to previously published studies, with some notable discernments. Our results apply to a general population of abdominoplasty candidates.

Seroma formation after abdominoplasty is a common complication, although the pathophysiology is still unclear. Disruption of lymphatic channels, “dead space” formation, extensive undermining and advancement of abdominal flaps, and shearing forces have all been implicated. Although seroma formation may be self-limited, it also may be associated with complications such as wound dehiscence, flap necrosis, and infection. Untreated seromas may form a bursa or pseudocyst, which may need to be surgically removed. Although surgical closed suction drains have been placed for decades for prevention of seroma formation, drains are associated with an increase in postoperative pain and have a risk of retrograde bacterial migration and infection.^61^ Strategies to reduce seroma rates have been proposed over the years, but few studies have reported on systematically evaluated outcomes. Although a previously published meta-analysis has reported decreased seroma formation rate with “preventative measures” compared to conventional abdominoplasty procedures with drains, the preventative measures group was a heterogenous mixture of PTS, Scarpa's fascia preservation, tissue adhesives, and abdominal flap dissection.^16^ In this study, the subgroup analysis of the effect of PTS vs drains demonstrated borderline differences (0.36 [0.13, 1.02]; *P* = .05). Previous meta-analyses also reported decreased seroma formation rates in PTS vs drains (odds ratio [OR] 0.36; 95% CI 0.19-0.70; *P* = .002; I^2^ = 9%).^1,2^ Additionally, there was no significant difference in postoperative seroma rates between PTS and PTS with drains (OR 1.03; 95% CI 0.30-3.54; *P* = .96; I^2^ = 0%). However, the failure of these studies to comply with AMSTAR 2 criteria limited the strength of their conclusions.

Interestingly, PTS was associated with a statistically significant decrease in reoperation rates (*P* = .05). This suggests that patients for whom surgery is approached with PTS may have a lower risk of reoperation. To determine the nature of this relationship with higher statistical power, it is critical to conduct further robust, homogenous studies to conclusively ascertain the superiority of PTS over drains in this context, through a future pooled meta-analysis. Additionally, our analysis highlights a need for more granular reporting of reoperative rates to permit more definite subtype analysis of reoperation, for example, with Clavien–Dindo classification.

A critical consideration when evaluating the optimal approach in abdominoplasty to minimize postoperative complications is liposuction and its impact. We addressed this by performing analysis on a subgroup of cohorts in which all patients underwent liposuction. In this subgroup analysis, interestingly, reduction in seroma rate with PTS alone vs drains was accentuated, with a greater reduction in risk ratio and reduced *P* values (RR 0.18; 95% CI 0.00-7.39; *P* < .00001). Additionally, liposuction alongside PTS compared to placing drains was associated with a statistically significant reduction in infection (RR = 0.16, *P* = .03; 95% CI 0.03 to 0.86) and dehiscence (RR = 0.11, *P* = .05; 95% CI 0.01 to 1.01). Our findings suggest that liposuction in abdominoplasty may be superior and synergistic with PTS over drains, indicating a potential way forward in the establishment of the superior surgical technique.

We found no statistically significant difference in postoperative infection rates between PTS and drain approaches. This is an important observation, because infection remains 1 of the most common postoperative complications and previous studies have claimed that PTS methodology increases the susceptibility to surgical site infections.^3,11^ Regarding dehiscence, our findings did not demonstrate a statistically significant reduction in the rate when PTS was compared with conventional surgical drainage, suggesting that both methods can be equally effective in preventing postoperative dehiscence. Furthermore, previous studies have described attempts to synergistically combine PTS with drains to maximize reduction of postoperative complications.^2^ However, our analysis demonstrated that PTS + drains (D) was not statistically significantly superior to PTS alone in the domains of seroma, hematoma, infections, reoperation rate, infection, or dehiscence.

There are inherent limitations in meta-analysis, and pooled results should be interpreted with caution due to heterogeneity among studies. Furthermore, it is not possible to eliminate all bias in retrospective studies. Additionally, further studies are required to compare the postoperative outcomes of PTS + D with PTS alone in the lipoabdominoplasty subgroup. Furthermore, individual-level data about BMI were not available or extracted. Postbariatric patients with massive weight loss or patients with BMI > 30 kg/m^2^ may introduce bias. Variables such as BMI, follow-up duration, methods of detecting seromas, compression garments, postoperative management strategies such as lymphatic massage, and heterogeneity of surgical technique such as number of progressive tension sutures or distance between sutures may all contribute to bias in data interpretation.

## CONCLUSIONS

In conclusion, our meta-analysis indicates potential benefits of PTS over conventional surgical drains for seroma and reoperation rate, with no significant difference observed in the rates of hematoma, infection, and dehiscence. An interesting area for further investigation is determining whether drains alone are superior to the PTS methodology with respect to reoperation rates. Indeed, investigation into the causes of the reduced reoperation rates with drains compared to PTS could offer valuable insights into the underlying mechanisms of action, potentially providing avenues for refining the technique and improving patient outcomes in abdominoplasty. This would be of the greatest benefit to the PTS-alone technique. We recommend that future research aims to reduce heterogeneity among studies to improve our ability to aggregate data on postoperative outcomes in abdominoplasty; incorporates larger sample sizes; and further explores the factors that could be influencing the observed outcomes. Given that patient satisfaction is a central tenet of surgical success in plastic surgery, the incorporation of patient-reported outcomes (PRO) in future studies is an important avenue for future research efforts. Finally, there is a need for larger, level 1 studies for evaluation of the safety and effectiveness of the drainless abdominoplasty technique, utilizing PTS vs drainage. Additionally, further delineation of larger seroma volumes (>80 mL) that might be indicative of negative patient prognoses would facilitate a more systematic classification of complications.

## Supplemental Material

This article contains supplemental material located online at www.aestheticsurgeryjournal.com.

## Supplementary Material

sjae171_Supplementary_Data
