# Supplementary material for: A Systematic Review and Meta-Analysis Evaluating the Surgical Outcomes of Progressive Tension Suturing Compared to Drains in Abdominoplasty Surgery
Source: Aesthet Surg J. 2024 Jul 30;45(1):71–83. doi: 10.1093/asj/sjae171 (PMC11634385; doi:10.1093/asj/sjae171)
Supplement: sjae171_Supplementary_Data [file sjae171_supplementary_data.zip › Supplementary Table 1.pdf]

*Supplementary Table 1 The quality of existing reviews assessed using the AMSTAR-2 criteria*

| AMSTAR 2 criterion | Our review | Li et al 2021 | Ho et al 2019 | Seretis et al 2017 | Jabbour et al 2016 |
|---|---|---|---|---|---|
| Item 1: Did the research questions and inclusion criteria for the review include the components of PICO? | Yes | Yes | Yes | Yes | Yes |
| Item 2: Did the report of the review contain an explicit statement that the review methods were established prior to conduct of the review and did the report justify any significant deviations from the protocol? | Yes | No | No | Partial Yes | No |
| Item 3: Did the review authors explain their selection of the study designs for inclusion in the review? | Yes | No | No | Yes | Yes |
| Item 4: Did the review authors use a comprehensive literature search strategy? | Yes | Partial Yes | Partial Yes | Partial Yes | Partial Yes |

| | | | | | |
|---|---|---|---|---|---|
| Item 5: Did the review authors perform study selection in duplicate? | Yes | Yes | Yes | Yes | Yes |
| Item 6: Did the review authors perform data extraction in duplicate? | Yes | No | Yes | Yes | No |
| Item 7: Did the review authors provide a list of excluded studies and justify the exclusions? | Yes | No | No | Partial Yes | No |
| Item 8: Did the review authors describe the included studies in adequate detail? | Yes | Partial Yes | Partial Yes | Yes | Partial Yes |
| Item 9: Did the review authors use a satisfactory technique for assessing the risk of bias (RoB) in individual studies that were included in the review? | Yes | No | No | Yes | Yes |
| Item 10: Did the review authors report on the sources of funding for the studies included in the review? | Yes | No | No | No | No |
| Item 11: If meta-analysis was justified | Yes | No | No | No | Yes |

| | | | | | |
|---|---|---|---|---|---|
| did the review authors use appropriate methods for statistical combination of results? | | | | | |
| Item 12: If meta-analysis was performed did the review authors assess the potential impact of RoB in individual studies on the results of the meta-analysis or other evidence synthesis? | Yes | No | No | Yes | Yes |
| Item 13: Did the review authors account for RoB in individual studies when interpreting/ discussing the results of the review? | Yes | No | No | No | Yes |
| Item 14: Did the review authors provide a satisfactory explanation for, and discussion of, any heterogeneity observed in the results of the review? | Yes | Yes | No | Yes | No |

| | | | | | |
|---|---|---|---|---|---|
| Item 15: If they performed quantitative synthesis did the review authors carry out an adequate investigation of publication bias (small study bias) and discuss its likely impact on the results of the review? | Yes | No | Yes | Yes | No |
| Item 16: Did the review authors report any potential sources of conflict of interest, including any funding they received for conducting the review? | Yes | Yes | Yes | Yes | Yes |
| Overall | High | Critically Low | Critically Low | Critically Low | Critically Low |
