# Supplementary material for: A Systematic Review and Meta-Analysis Evaluating the Surgical Outcomes of Progressive Tension Suturing Compared to Drains in Abdominoplasty Surgery
Source: Aesthet Surg J. 2024 Jul 30;45(1):71–83. doi: 10.1093/asj/sjae171 (PMC11634385; doi:10.1093/asj/sjae171)
Supplement: sjae171_Supplementary_Data [file sjae171_supplementary_data.zip › Supplementary Table 2.pdf]

Supplementary Table 1 Search strategy supplementary figures – example search strategy used for MEDLINE with Boolean operators and functions detailed. Similar strategies were employed for EMBASE, CENTRAL and Web of Science.

1	exp Abdominoplasty/
2.	Abdominoplasty.ti,ab,kw.
3.	"tummy tuck".ti,ab,kw.
4.	1 or 2 or 3
5.	exp Drainage/
6.	drain*.ti,ab,kw.
7.	5 or 6
8.	(progressive adj2 tension* adj2 sutur*).mp. [mp=title, book title, abstract, original title, name of substance word, subject heading word, floating sub-heading word, keyword heading word, organism supplementary concept word, protocol supplementary concept word, rare disease supplementary concept word, unique identifier, synonyms]
9.	4 and 7 and 8
10.	exp Hematoma/
11.	exp Seroma/
12.	exp Surgical Wound Dehiscence/
13.	Infections/
14.	10 or 11 or 12 or 13
15.	9 and 14
