# Supplementary material for: A Systematic Review and Meta-Analysis Evaluating the Surgical Outcomes of Progressive Tension Suturing Compared to Drains in Abdominoplasty Surgery
Source: Aesthet Surg J. 2024 Jul 30;45(1):71–83. doi: 10.1093/asj/sjae171 (PMC11634385; doi:10.1093/asj/sjae171)
Supplement: sjae171_Supplementary_Data [file sjae171_supplementary_data.zip › Supplementary Table 3.pdf]

	Journal	Year	Country	Design	Type	No. of Centres	Ethics	Funding	Size	Age ± SD	Female: Male	Inclusion/Excl-usion criteria	PTS technique	Drains technique	Duration of follow up
N. Agochu-kwu-Nwubah et al.	Aesthetic surgery journal	2021	USA	Retrosp-ective review	Chart review	1	Guided by principles according to the Declaration of Helsinki. Demographic data were collected on patients.	NF	351	49.6	340:11	All patients who underwent a flap procedure by the senior author (C.P.) over 3 years (January 2016-December 2018) at a single in-stitution (Aesthetic Center for Plastic Surgery, Houston, TX). These were consecutive patients, and flap procedures included abdominoplasty, torsoplasty, brachioplasty, lower body lift, and medial thigh lift.	For all flap procedures PTS was used - a 2-0 polydioxanone suture (PDS) used for a running PTS in the midline along the linea alba from the xiphoid to the point immediately above the umbilicus.	No drains used.	3 years
P. Andrades et al	Plastic & Reconstru-ctive Surgery (PRS)	2007	Chile	RCT	Double blinded	1	Department of Surgery Ethical Committee of the University of Chile Clinical Hospital	NR	55	Group 1: 40.7 ± 5.8<br><br>Group 2: 38.5 ± 4.6<br><br>Group 3: 40.1 ± 9.5<br><br>Group 4: 39.7 ± 6.1<br><br>Total = 39.8 ± 6.5	100:0	Non-overweight female patients with an ante-rior abdominal wall deformity type IV, without comorbidity.	If PTS was used, 30 to 40 separate fixation and advancement sutures were placed between the flap and the fascia using an absorbable material.	If drains were used, two closed suction drains were placed with exit at the pubis 3 cm below the incision. A 150-cc flank liposuction with a previous 80-cc infiltration was carried out to improve contouring results in all patients. No liposuction of the dorsum or	6 months

														any additional surgical procedures were performed.	
J. W. Antonetti et al	Aesthetic surgery journal	2010	USA	Retrosp-ective review	NR	1	NR	NF	517	Female: 36.7 ± 8.6<br><br>Male: 36.5 ± 12.8	508:9			No drains used.	
H. L. Arantes et al	Aesthetic plastic surgery	2010	Brazil	Retrosp-ective study	Case control	1	NR	NF	60	NR	50:0	Women who underwent classic abdominoplasty with adhesion sutures between January 2006 and December 2007 - conducted by the same team of surgeons at a single institution. The recorded data included the name, date of surgery, technical details, postoperative complaints, clinical incidence of seromas, time of drain removal, and long-term follow-up evaluation.	The patient was placed in a bent position to reduce tension on the abdominal flap. The adhesion suturing was performed intermittently using Vicryl 2-0 along the median line and equidistant from this line on either side of the abdomen, with approximately 4 cm between the stitches.	Classic abdominoplasty with adhesion sutures and the inclusion of suction drains.	6 months
A. Arnaout et al	British Journal of Surgery	2020	UK	Retrosp-ective study	NR	1	NR	NF	53	NR	NR	The use of progressive tension barbed sutures in the closure of a deep inferior epigastric perforator (DIEP) flap donor sites.	Donor sites were closed with bilateral progressive tension barbed sutures (3-0 V-LocTM Wound Closure Device - Medtronic (UK)).	No drains used.	NR
R. A. Baxter	Aesthetic Plastic Surgery	2001	USA	Retrosp-ective study	Review	1	NR	NF	111	NR	NR	Patients who underwent either a standard abdominoplasty or mini-	Absorbable sutures such as OO polydioxanone were used. After insetting	No drains used.	1-42 months

												abdomino-plasty. The operative technique was recorded regarding use of anchor sutures, and the postoperative record was reviewed with any complica-tions noted.	the umbilicus at the proper level, the an-chor sutures were continued in sequence. Midline progressive tension sutures were also used to help create an attractive groove.		
M. Bromley et al	Aesthetic Plastic Surgery	2018	Brazil	RCT	Single blind, prospec -tive, compar-ative	1	The study was approved by the Brazilian Investigation Ethical Committee Board (http://plataformabrasil .saude. gov.br) and conducted in accordance with the Declaration of Helsinki.	NF	63	Group 1: 46.7<br><br>Group 2: 45.6<br><br>Group 3: 48.3	49:14:00	Inclusion criteria were patients aged between 18 and 60 years; those with a type 3 abdominal Pitanguy deformity (lipodystrophy, abdominal flaccidity, and rectus diastasis with or without a previous scar) and Matarasso type 4 anterior abdominal deformity (severe laxity and complete diastasis); and those willing to undergo abdominoplasty without liposuction. Exclusion criteria were body mass index (BMI), unstable clinical pathologies such as type II diabetes or uncontrolled hypertension, postbariatric surgery, hematologic diseases, surgical planning of any	PTS were applied using 3-0 Vicryl (Fig. 5) after the excess abdominal flap was removed. The sutures were applied between the aponeurosis and the abdominal flap. The wounds were closed in layers using Nylon 3-0, 4-0 and Monocryl 3-0, after which Steristrips (Skin Closure, 3M, St. Paul, MN, USA) were applied.	Two negative pre sure drains connected to a single chamber, Hemovac drains (Zimmer, Dover, OH, USA), were positioned in all patients through stab incisions in the pubic area. The drains were left until the total volume was less than 30 ml within 24 h.	90 days

												combined procedure (e.g., mammoplasty, facelift, and rhinoplasty), secondary abdomino-plasties, lipoabdominopl-asties, flor de lis abdominoplasti-es, circumferential abdominoplasti-es, and patients with restricted follow-up.			
J. V. Cucchiaro et al	Plastic and Reconstru-ctive Surgery-Global Open	2017	Argentina	Retrosp-ective study	Review	1	Followed the principles of the Declaration of Helsinki.	NF	290	38	272:4	Exclusion criteria were as follows: patients with a body mass index (BMI) of over 30 kg/m2, postbariatric patients, patients who had lost more than 25 kg/m2 before surgery, and patients who were pregnant.	The PTSs were placed with the patient in Fowler's position. 3 Vicryl 2-0 running sutures were placed in Scarpa's fascia, 1 in the supra-umbilical midline and 2 in the infra-umbilical paramedian. These sutures served to pull down the abdominal flap, decreasing scar stress in the supra-pubic and umbilical areas.	No drains used.	2 years
S. Gallagher et al	Annals of Plastic Surgery	2018	USA	Retrosp-ective study	Chart review	1	Approved by the Indiana University Institutional Review Board. To prepare for surgery, all candidates were seen by a mental health professional and assessed for suitability for surgery with guidance of the World Professional Association for Transgender Health standards of care.	NF	153	26.9	NR	Most patients presenting for this technique had too much skin and breast tissue to undergo less invasive "keyhole" tech-niques. Patients presenting for gender confirmation surgery who did qualify for	Using a 1-0 Vicryl suture with the largest tapered needle available, multiple interrupted sutures were placed from the superficial fascia of the superior flap. Tension was placed on the superior flap, and a bite of	No drains used.	9 months

											minimal scar techniques were excluded from this study.	the more caudal pectoralis fascia was taken, and the suture tied down.		
Daniel J Gould et al	Aesthetic Surgery Journal	2018	USA	Retrosp -ective study	Cohort study	1	Conducted in accordance with the Helsinki and Nuremberg principles with written consent from all included patients.	NF	619	PTS group: 45.1<br><br>Drain group: 44.2	597:22:00	Groups compared included classic drain-based abdominoplasty (n = 320) and PTS abdominoplasty (n = 299). Four patients were excluded from the study, because there was incomplete information on the location or volume of liposuction. Also, those groups were subdivided into groups with and without liposuction.	NR	NR	Range PTS group: 0-69 months<br><br>Mean Drain group: 0-80 months
S. Khan et al	Annals of Plastic Surgery	2006	USA	Retrosp -ective study	Case control	1	NR	NR	49	37.3 ± 7.7	47:2	All patients who underwent an abdominoplasty procedure by the senior author (PAW) during a 5-year period (2000–2004) were considered eligible for inclusion in the study. Patients were identified by querying the surgical database at the Charlotte Plastic Surgery Center.	The patient is then placed in a flexed position to decrease tension on the abdominal flap. PTS are then placed intermittently along the midline raphe at intervals of approximately 1 cm utilizing 0 Vicryl (Ethicon) suture. The sutures are placed from the superficial (Scarpa's) fascia to the deep fascia. The flap is progressively advanced inferiorly with the placement of each suture	A closed suction drain was placed on either side of the abdomen. Drains are left in place until drainage from each is less than 30 mL/24 hours for 2 consecuti-ve days.	5.42 ± 3.64 months

U. D. Khan et al	European Journal of Plastic Surgery	2011	UK	Retrosp -ective study	Clinical review	1	NR	NF	121	Group A: 37 ± 6.7<br><br>Group B: 39.5 ± 9.5<br><br>Group C: 40.6 ± 10.9	NR	Group A included patients with abdominoplasty and liposuction of flanks. Group B included with abdominoplasty , liposuction of flanks, and progressive tension sutures. Group C had abdominoplasty , limited supraumbilical undermining, progressive tension sutures, liposuction of the abdominal wall and flanks.	Progressive tension sutures were applied only in groups B and C using 2-0 Vicryl. The umbilicus was isolated on its pedicle in the sample studied, and rectus plication was performed where necessary.	NR	NR
L. H. Maclas et al and W. Grant Stevens	Aesthetic surgery journal	2016	USA	Retrosp -ective study	Analysis	1	This study was not IRB approved, but prior written consent was obtained from all patients who participated in this study. The study was conducted in accordance with the guidelines of the Declaration of Helsinki.	NF	451	Drains: 44.3<br><br>PTS: 44.1	433:18	"Mini" abdominoplasties, where the flap was only elevated to the umbilicus rather than to the xyphoid, and circumferential body lifts were excluded from this study. Additionally, secondary abdominoplasties were also excluded.	0-vicryl quilting sutures were placed in a progressive fashion along the midline. The PTS sutures were placed in 2 to 3 points both supraumbilical and 2 infraumbilical in the mid-sagittal plane while providing aggressive inferior advancement of the abdominal flap. Additional lateral quilting sutures were placed in the infraumbilical region; usually 3 to 4 sutures per side, totalling 10 to 12 sutures in total. The incision was then closed in a layered fashion.	Two Jackson-Pratt drains were placed exciting out the skin in the suprapubic region and the incision closed in a layered fashion.	Drain (mean): 9.3 months<br><br>PTS (mean): 6 months

A. T. Mohan, et al.	Journal of plastic, reconstructive & aesthetic surgery	2015	USA	Retrospective study	Review	1	This study was approved by the Institutional Review Board (IRB) at the Mayo Clinic, Rochester, Minnesota.	NF	93	Drains: 50.2 ± 8.9<br><br>No drains: 47.9 ± 7.6	NR	Any women who underwent a free TRAM or had any flap or breast complications during their inpatient stay were excluded. Women who had a bilateral procedure with a DIEP but may have had one side converted to a muscle-sparing (MS-TRAM) were included.	The progressive tension suture placement was carried out using 2/0 Quill PDO barbed suture (Angiotech Pharmaceuticals, Inc., Vancouver, BC, Canada), which is a barbed double arm suture. Central quilting sutures were first placed above the umbilicus with 2/0 Vicryl, then two "V" patterns were created in each hemi-abdomen using the 2/0 Quill, by a continuous suturing technique to secure the abdominal flap onto the anterior abdominal fascia. At the distal points, remaining suture was used to close Scarpa's fascia at the incision line towards the midline. Closure using B-PTS was carried out from proximal to distal, either in short segments bilaterally or simultaneous closure of both sides by two surgeons.	Standard closure was performed as a layered closure of Scarpa's, deep dermis and subcutis at the incision line using a monofilament absorbable suture: PDS for Scarpa's fascia and 3/0 Monocryl for deep dermis and Monoderm for skin. Two (but up to four) suction drains were used.	NR
D. Parvizi et al.	Obesity Surgery	2015	Austria	Retrosp-ective study	Multiple regression analysis	1	Prior approval for the study had been obtained from the institutional review board.	NF	205	42	182:23	Patients who had undergone abdominal dermolipectom-y (defined as	NR	NR	5.94 years

												undermining of skin to the level of the xiphoid with/without plication of the rectus fascia) over a 6-year period (2001–2006). All the operations were performed under general anesthesia by plastic surgeons, with little variation in each individual surgeon's operative technique.			
J. F. Pascal et al	Aesthetic Plastic Surgery	2008	France	Retrosp -ective study	Chart review	1	NR	NR	235	NR	NR	NR	NR	NR	NR
Pollock et al	Clinics in Plastic Surgery	2004	USA	Retrosp -ective study	Review	1	NR	NR	65	42	NR	NR	The wound is closed in layers with 3-0 Vicryl in the deep dermis and 4-0 PDS running subcuticular in the skin.	No drains used.	15 months
Pollock et al	Aesthetic surgery journal	2012	USA	Retrosp -ective study	Chart review	1	NR	NF	597		587:10	All abdominoplasti -es performed by the authors during a 12-year period (January 1998 through December 2009).	PTS were placed from the superficial to the deep fascia as the abdominal flap is advanced.	No drains used.	13.7 months
A, Rosen et al	Aesthetic Surgery Journal	2019	USA	Retrosp -ective study	Multicen -tre analysis	2	Written informed consent for publication of results was signed by all patients in this consecutive series, in adherence with the Committee on Publication Ethics guidelines.	U	445	Centre 1: 45<br>Centre 2: 45	431:14	Patients who underwent abdominoplasty with absorb-able, running, barbed, progressive tension sutures (Quill knotless tissue-closure device; Surgical Specialties Corporation, Reading, PA) since July 2007 were included in this 10-year retrospective	For PTS, a surgical marker was used to mark a vertical grid pattern on the abdominal wall that was imprinted onto the cutaneous flap by pressing the flap against the fascia. Rosen now begins with a single-armed midline suture and then places a double-armed	No drains used.	6 months

												analysis, with the last included procedure occurring in November 2018.	paramedian suture, to create 2 additional rows on each side. Then, both operators simultaneously proceed with PTS.		
Marcos Sforza, et al	Aesthetic Surgery Journal	2015	UK	Retrosp -ective study	Analysis	1	This clinical audit followed the Declaration of Helsinki guidelines and a written consent for the outlined procedure was obtained from all patients.	NF	414	41.2	414:0	414 female patients, undergoing abdominoplasty surgery with flank lipo-contouring between January 2007 and December 2011. All surgeries were performed in a private UK hospital. Combined abdominoplasty and lipo-contour patients were selected due to their increased risk of seroma formation compared to abdominoplasty only patients, allowing us to extensively evaluate quilting suture efficacy.7 Prior to participation in the study, written informed consent was acquired from all patients included in the study.	Quilting sutures used to attach an undermined flap to the abdominal wall fascia.	Drains were introduced through the pubic area in all three groups, using size 14 vacuum system drains.	Up to one year
Patricio Andrades, M.D. et al	Plastic and reconstruc tive surgery	2007	Chile	RCT	Prospec -tive, double blind trial	1	This study was approved by the Department of Surgery Ethical Committee of the University of Chile Clinical Hospital	U	60	Control Group: 40.7 ± 5.8<br><br>PTS group:	60:0	Non-overweight female patients with an ante-rior abdominal wall deformity type IV without comorbidity,	If progressive tension sutures were used, 30 to 40 separate fixation and advancement sutures were	If drains were used, two closed suction drains were	6 months

												analysis, with the last included procedure occurring in November 2018.	paramedian suture, to create 2 additional rows on each side. Then, both operators simultaneously proceed with PTS.		
Marcos Sforza, et al	Aesthetic Surgery Journal	2015	UK	Retrosp -ective study	Analysis	1	This clinical audit followed the Declaration of Helsinki guidelines and a written consent for the outlined procedure was obtained from all patients.	NF	414	41.2	414:0	414 female patients, undergoing abdominoplasty surgery with flank lipo-contouring between January 2007 and December 2011. All surgeries were performed in a private UK hospital. Combined abdominoplasty and lipo-contour patients were selected due to their increased risk of seroma formation compared to abdominoplasty only patients, allowing us to extensively evaluate quilting suture efficacy.7 Prior to participation in the study, written informed consent was acquired from all patients included in the study.	Quilting sutures used to attach an undermined flap to the abdominal wall fascia.	Drains were introduced through the pubic area in all three groups, using size 14 vacuum system drains.	Up to one year
Patricio Andrades, M.D. et al	Plastic and Reconstru -ctive surgery	2007	Chile	RCT	Prospec -tive, double blind trial	1	This study was approved by the Department of Surgery Ethical Committee of the University of Chile Clinical Hospital	U	60	Control Group: 40.7 ± 5.8<br><br>PTS group:	60:0	Non-overweight female patients with an ante-rior abdominal wall deformity type IV without comorbidity,	If progressive tension sutures were used, 30 to 40 separate fixation and advancement sutures were	If drains were used, two closed suction drains were	6 months

										38.5 ± 4.6<br>Drain group: 40.1 ± 9.5<br>PTS & Drains: 39.7 ± 6.1<br>Total: 39.8 ± 6.5		were included in the study.	placed between the flap and the fascia using an absorbable material.	placed with exit at the pubis 3 cm below the incision.	
M. Vernier-Mosca et al	Annals of Aesthetic Plastic Surgery	2021	France	Retrosp -ective study	Retrosp -ective analysis	1	NR	NR	215	Draina -ge group: 42.2 ± 10.2<br>No drain- age group: 46.3 ± 11.9	204:11	From January 1 2017 to December 31 2019, all abdominoplasti es with transposition of the umbilicus were included in the study.	The cure diastasis was carried out, if necessary, using a PDS® (Ethicon) loop 1.0 overlock of the xiphoid process to the pubic region. The padding was made medially by an overlock of V-Lock® 3.0 notched thread, creating a tension suture progressive from the xyphoid process to the suprapubic subcutaneous tissue, between the fascia superficialis and the aponeurosis. Laterally, the padding was carried out either by points deep separations of Vicryl® (Ethicon) 2.0 (3 to 4 points arranged symmetrically on both sides other side of the median line), or by two	Two Blake® drains, charriere 10, were connected to suction bottles low pressure and were put in place before closing. The outlet orifices were located under the lower scar at the level of the inguinal folds.	9.9 months

overlocks as
previously
described
opposite
of the lateral
edges of the
rectus muscles

Study Characteristics

	Title (red=excluded)	Author/year (green =	Study design	Groups (D or PTS or l	Number of patients	Liposuction % (% of	Age Y	BMI	Gender	PTS technique	Electrocautery	Scalpel	Ultrasonic scalpel	Glue	PTS sutures used	PTS interrupted or cc	Scarpa (Y= preserve	Drainage technique	Drain total output	Drain removal	Lipoabdominoplasty	Seroma (n)	SEROMA %	Method of seroma ca	Haematoma (n)	Haematoma %	Flap Necrosis (n)	Flap necrosis %	Re-operation (n)
1	360degree Abdominoplasty: A Circumferential Approach to Contouring of the	Amalfi A. N., Koenig W.J., Skibba K.E.H./ 2021	expert opinion	PTS and lipo																									
2	A case for the safety and efficacy of lipoabdominoplast y: A single surgeon	J. Weiler; P. Taggart; K. Khoobehi / 2010	Retrospective review	PTS and lipo	173	1	41.53	26	F: 171 M: 2	NR	Y	Y	N	N	NR	Continuous	N	N	N	N	Lipoabdominoplast y	6	0.034	NR	NR	NR	2	0.011	14
3	A Multiple Regression Analysis of Postoperative Complications	Parvizi, D.; Friedl, H.; Wurzer, P.; Kamolz, LP.; Lebo, P.; Tuca, A.; Rappl, T.; Wiedner, M.;	Retrospecitve	D	205		42 (20–77)	29 (19-62)										vacuum drains		<25ml		10	0.05		15	0.07			
4			Analysis																										
5	A prospective, randomized-contr olled pilot study comparing closed suction versus																												
6	Abdominoplasty and seroma: A prospective randomised study comparing scalpel	Marsh, D.J.; Fox, A.; Grobbelaar, A. O., Chana J.S /2014	Prospective randomised study	D	102	0.38	45.4	group 1 = 27.1 group 2 = 27.6	F:97 M: 5	N	Y (n=58)	Y (n=44)	N	N	N	N	Y	two redivac drains size 10	group 1 = 133.4 ; group 2 = 86	48h post-op	Standard adominoplasty	19	0.193	clinical	3	0.0306	NR	NR	NR
7	Abdominoplasty in the Obese Patient: Risk versus Reward.	Joseph Batac; M. Hamade; H. Hamade; L. Glickman/ 2019	Retrospective	D	83	0.831	42.9	28.1	F:81 M: 2	N	Y	N	N	N	N	N	N	vacuum drains	NR	9 days	Standard adominoplasty / Lipoabdominoplas ty	17	0.204	clinical	2	0.024	NR	NR	NR
8			Analysis																										
9	Abdominoplasty With Progressive Tension Closure Using A Barbed Suture Technique.	J. P. Warner; K. A. Gutowski / 2009	Prospective study	PTS	58	0.5517	42		F: 58	bidirectional barbed suture	Y	Y	N	NR	polydioxanone absorbable monofilament		N	N	N	N	Full abdominoplasty	1	0.017	clinical	0	0	0	0	0
10	Abdominoplasty With Scarpa Fascia Preservation: Prospective	Pisco, A; Rebelo M; Peres, H; Costa-Ferreira,A; 2020	Prospective study	D	73	NR	40.5	25	F:73	N	Y	NR	NR	NR	N	N	Y	vacuum drains	186,5	<50ml/day	Lipoabdominoplast y	9	0.123	clinical	3	0.041	3	0.041	1
11	Abdominoplasty without Drains or Progressive Tension Suturing.	F. A. Ali Khan; M. Fatima/ 2021	Retrospecitve	Nothing	100	0.56	40	25.1	F:89 M: 11	N	Y	Y	NR	N	N	N	N	N	N	N	Standard adominoplasty	6	0.06	clinical	2	0.02	NR	NR	NR
12			Analysis																										
13	Abdominoplasty, seroma formation and duration of hospitalisation: comparative	U. D. Khan/2019	Retrospecitve	Group A(27) - Ambulatory cases	112	1	Group A: 36.1 ± 6.98	Group A: 23.8 ± 3.02		Flap medially & inferiorly	NR	NR	Y	NR	0-Vycril	Interrupted	N	vacuum drains size 10	Mean in first 24 hrs:	24-48h post-op	Lipoabdominoplast y	Group A: 0	Group A: 0%	clinical	Group A: 0	Group A: 0%	NR	NR	Group A: 0
14			Analysis	Group B (68) - Hospital stay of 1night			Group B: 40.6 ± 11.36	Group B: 27.4 ± 5.34		displaced and two rows of PTS									Group A: 26.9 ± 17.24 cc			Group B: 0	Group B: 0%		Group B: 0	Group B: 0%			Group B: 0
15				Group C (17) - Hospital stay of 2 days			Group C: 38.8 ± 9.66	Group C: 27.0 ± 4.06		placed, 2-3 on each side.									Group B:52.1 ± 58.8 cc			Group C: 0	Group C: 0 %		Group C: 0	Group C: 0 %			Group C: 0
16																			Group C: 80.1 ± 44.55 cc										
17	Anchor-Line Abdominoplasty with Scarpa Fascia Preservation in Postbariatric	H C B Inforzato; E B Garcia; J C Montano-Pedroso; L A Rossetto; L M Ferreira/ 2020	Prospective randomized study	D	42	0	37.9	27.4		N	Y	Y	NR	NR	N	N	Group A (21): Y (preserved) Group B :21 N (not preserved)	vacuum drains	group A = 441cc ; group B = 264cc	<30ml/day	Standard adominoplasty	6	0.142	clinical /confirmed by ultrasound	0	0	0	0	0
18	Closed Incision Negative-Pressure Therapy (ciNPT) Reduces Minor Local	Silvio Abatangelo1 & Elisabetta Saporiti2 & Giorgio Giatsidis3	Retrospecitve	D	11	0	36.09	34	F:6 M:5	N	NR	NR	NR	NR	N	N	NR	vacuum drains	NR	NR	Standard adominoplasty	1	0.09	clinical	1	0.09	2	0.18	1
19			Analysis																										
20	Comparative study of wound complications: Isolated panniculectomy	Alla Y. Zemlyak; Paul D; Colavita; Sofiane El Djouzi;	Prospective study	D		NO	47.6	41.1	F: 39 M: 3	N	NR	NR	NR	NR	N	N	NR	vacuum drains	NR	<30ml/day	Standard adominoplasty	5	0.128	clinical	1	0.026	NR	NR	4
21		Amanda L. Walters; Logan Hammond; Brandon Hammond; Victor B. Tsirline;																											
22		Stanley Getz; B. Todd Heniford/ 2012																											
23	Comparison of energy-based tissue dissection techniques in abdominoplasty: A	D. Duscher; M. M. Aitzetmueller; Jing-Jing Shan; R. Wenny; E. A. Brett; C. J. Staud; D.	Prospective randomized study	D	57	0	38	26.8	NR	N	Y	Y	Y	NR	N	N	NR	gravity drains	from 50ml to 700ml	<30ml/day	Standard adominoplasty	7	0.0399	clinical /confirmed by ultrasound	4	0.0228	NR	NR	0
24	Continuous Negative Pressure Drain is Associated with Better Outcome: A	Ting Jiang, Jun Li , Jun Ren / 2019	Randomized prospective trial	D	58	NO	47.5± 7.5	NR	F	No PTS	NR	N	N	NR	NR	NR	NR	Continuous negative pressure drainage vs suction drain	13.6 ± 4.5 mL intervention group and 8.0 ± 4.0 mL control group	less than 5 in 2 consecutive days	NR	1 in intervention group and 6 in control group	3.4% and 20.6% control group,	Clinical	NR	NR	4 in control group	13.7% in Control group	NR
25	Decrease in seroma rate after adopting progressive tension sutures	Luis H Macias, Edwin Kwon, Daniel J Gould, Michelle A Spring, W Grant Stevens	Retrospecitve	PTS: 127	451	liposuction in 186 patients	PTS: 44.1 (22-72) D: 44.3 (19-71)	PTS: 26 (18-37.9)	433 (96%) female and 18 (4%) male	2-3 supraumbilical & 2 infra-	Y	N	N	NR	0-vicryl quilting sutures	Continuous	NR	2 Jackson-Pratt drains	NR	NR	The elevation was carried supra-umbilically in a narrow dissection over	PTS: 3	PTS: 2% D: 9%	Clinical	PTS: 0 D: 3	PTS: 0%	0	0	PTS: 22 D: 33
26			Analysis	D: 324				D: 25.4 (17-43)		umbilical along the mid-								in supra-pubic region				D: 28				D: 1%			
27										sagital plane																			
28	Do Abdominal Binders Prevent Seroma Formation and Recurrent Diastasis	Maria Roberta Cardoso Martins, Betina Zimmermann Fontes de Moraes,	Randomized trial	D	34	NO	34.8	24.8	F	No PTS	Y	N	N	NR	NR	NR	NR	Penrose drain 1 cm in diameter was inserted laterally through the incision	NR	24h	A suprapubic incision was made and extended laterally to the anterior superior			USS	NR	NR	NR	NR	NR

Study Characteristics

	Title (red=excluded)	Author/year (green =	Study design	Groups (D or PTS or	Number of patients	Liposuction % (% of	Age Y	BMI	Gender	PTS technique	Electrocautery	Scalpel	Ultrasonic scalpel	Glue	PTS sutures used	PTS interrupted or co	Scarpa (Y= preserve	Drainage technique	Drain total output	Drain removal	Lipoabdominoplasty	Seroma (n)	SEROMA %	Method of seroma ca	Haematoma (n)	Haematoma %	Flap Necrosis (n)	Flap necrosis %	Re-operation (n)	Re-operation (%)	Followup
28	Do Abdominal Binders Prevent Seroma Formation and Recurrent Diastasis	Maria Roberta Cardoso Martins, Betina Zimmermann Fontes de Moraes,	Randomized trial	D	34	NO	34.8	24.8	F	No PTS	Y	N	N	NR	NR	NR	NR	Penrose drain 1 cm in diameter was inserted laterally through the incision	NR	24h	A suprapubic incision was made and extended laterally to the anterior superior			USS	NR	NR	NR	NR	NR	NR	6 months
29	Drainless abdominoplasty Using Progressive Tension Sutures.																														
30	Effect of quilting sutures on seroma formation post-abdominoplasty.	Lauren Ovens & Mark A. Pickford / 2009	retrospective study	D: 34 PTS 40	76	NO	25-76	NR	F	Suture deep surface of the abdominal flap to the deep fascia of the	Y	N	N	NR	2-0 vicryl	NR	NR	vacuum drains	NR	NR	Standard adominoplasty	PTS: 6 D: 10	PTS:17.6% D:25%	Clinical	PTS: 3 D: 4	PTS: 8.8% D10%	PTS: 0 D: 1	PTS: 0 D: 2.5%	PTS: 0 D:4	PTS: 0 D:10%	NR
31										abdominal wall under downward and medial tension																					
32	Efficacy of intraoperative hypertonic saline irrigation in seroma prevention	Mateusz Zamkowski, Maciej Śmietański / 2023	Cohort study																												
33	Evaluation of the Number of Progressive Tension Sutures Needed to Prevent	Miluska Bromley, Walter Marrou & Luiz Charles-de-Sa /2018	RCT	Variants of PTS+D - group 1, classic abdominoplasty without the use of PTS and drains;	63	NO	1: 46.7 2: 45.6 3: 48.3	1: 27.8 2: 27.5 3: 26.9	F: 49 M: 14	group 1, classic abdominoplasty without the use of PTS and drains; group 2,	Y	N	N	N	3-0 vicryl	NR	NOT PRESERVED	Two negative pres-sure drains connected to a single chamber, Hemovac drains	NR	NR	Standard adominoplasty	5	0.11	Clinical + USS	NR	NR	NR	NR	NR	NR	90 days
34	Expanding the Progressive Tension Suture Concept: A Study of Its Utility in	N. Agochukwu-Nwubah and C. Patronella 2021 Aesthetic Surgery	Retrospective study	PTS	305 (PTS)	57% (174/305)	46 (25-68) (age (range))	24.9 (17.8-33.8) (BMI (range))		Interrupted sutures (Pollock and Pollock). For PTSs in abdominoplasty (Figure 1; Video 1),	NR	NR	NR	NR	2-0 polydioxanone suture (PDS)	Interrupted	NR	No drain	N	No drain	Standard adominoplasty	0	0	Clinical	3	0.984 % (3/305)	4	0.012	1	0.006	3 months to 3 years
35	Fat necrosis and dermolipectomy [23].																														
36	Fibrin sealant and lipoabdominoplasty in obese grade 1 and 2 patients.	Amr Abdel Wahab Mabrouk, Hesham Aly Helal, Soha Fathy Al Mekkawy, Nada Abdel Sattar	Randomized prospective	Group B: D Group A: D + Fibrin Glue	A: 30 ; B: 30	0.5	38.5±9.5 years) in group A, 37.8±9.1 years) in group B	32.6 kg/m2 in group A ; 31.5 kg/m2 in group B	F: 58; M: 2	No PTS	NR	Y	NR	Y	N	N	Y	2 silicone suction drains	NR	removed when the daily drainage volume was less than 30 mL, which was on the 5th day	Standard adominoplasty	A: 1; B:11	3% group A ; 37% group B	USS	NR	NR	A: 0; B:1	B: 3%	NR	NR	21 Days
37	Full abdominoplasty with circumferential	Cárdenas-Camarena Lázaro, Laguna-Barraza Victor / 2007	Retrospective study	2007	D	Y	36	15 patients (12%) were considered to be at	F	No PTS	NR	NR	NR	NR	NR	NR	NR	soft silicone negative suction drain	NR	NR	Standard adominoplasty	0	0	Clinical	0	0	0	0	0	0	6 months
38								their ideal weight, 95 patients (78%) were overweight																							
39								(BMI between 25 and 30 kg/m2), and 12 (10%) were																							
40								obese (BMI higher than 30 kg/m2).																							
41	Global Prevalence and Preferences of Progressive Tension Suture Usage in																														
42	Health technology assessment through the six sigma approach in abdominoplasty:		C. Ricciardi, A. Gubitosi, G. Lanzano, S. Parisi, E. Grella, R. Ruggiero, S. Izzo,																												
43	High volume, drainless lipoabdominoplasty with progressive tension sutures																														
44	Ligating perforators in abdominoplasty reduces the risk of seroma.	J.M Skillman, M. R. Venus, P. Nightingale, O. G. Titley & A. Park; 2014	Prospective cohort study	D	90	NO			F: 85 M: 5		Y							Suction drains		Less than 30ml in last 24h	Standard adominoplasty	14	0.234	Clinical							
45	Lipoabdominoplasty without drains or progressive tension sutures: An analysis of 100	S. Epstein; M. A. Epstein; K. A. Gutowski/2015	Retrospective study	PTS	100	1	40	25.1	F: 97 M: 3	double layer running suture	NR	NR	NR	NR	Barbed absorbable suture	Continuous	N	N	N	N	Standard adominoplasty / extendend / circumferencial	5	0.05	Clinical	2	0.02	NR	NR	3	0.03	3 months
46	Meta-analysis on the comparative efficacy of drains, progressive tension sutures																														
47	Progressive Tension Sutures Eliminate the Need for Drains in Body Contouring																														
48	Progressive tension sutures in the prevention of postabdominoplasty seroma: A	P. Andrades; A.Prado; S.Danilla; C. Guerra; S. Benitez; S.Sepulveda;	Prospective double blind clinical trial	Group A(no PTS or D) - 10	55	1	39.8	26.4	F:100	30 - 40 separate fixation	Y	NR	NR	NR	Absorbable suture	Interrupted	NR	vacuum drains	NR	7 days	Standard adominoplasty	Group A : 10 Group B: 13 Group C: 13 Group D: 14	Group A : 100% Group B: 87% Group C: 87% Group D: 93%	Clinical / Ultrasound	0	0	0	0	NR	NR	6 months
49				Group B (PTS) - 15																											
50				Group C (D) - 15 Group D (D + PTS) - 15																											
51	Reduced Seroma Risk in Drainless Abdominoplasty Using Running Barbed Sutures: A	A, Rosen. K, Gutowski. T. Hartman/ 2019	Retrospective analysis	PTS	445	N	45	26.4	F: 431 M: 14	six row continuous pts	NR	NR	NR	NR	Polypropilene and PDO	Continuous	N	N	N	N	Standard adominoplasty / circumferencial	21	0.047	Clinical	7	1.6	NR	NR	0	0	6 months
52	Reducing seroma in outpatient abdominoplasty: Analysis of 516 consecutive	John W Antonetti, Alfred R Antonetti 2010	Retrosprctive study	PTS	517	Y	36.6		F:508 M:9	Several rows Interrupted	NR	NR	NR	NR	2-0 Vicryl	Interrupted	NR	vacuum drains													

Study Characteristics

	Title (red=excluded)	Author/year (green =	Study design	Groups (D or PTS or	Number of patients	Liposuction % (% of	Age Y	BMI	Gender	PTS technique	Electrocautery	Scalpel	Ultrasonic scalpel	Glue	PTS sutures used	PTS interrupted or cc
	consecutive															
53	Risk of seroma with simultaneous liposuction and abdominoplasty and the role of	U. D. Khan/2007	Retrospective study	Group A (D) - 96 Group B (D + PTS) - 50	146	0.53	Group A - 39.5 Group B - 37	Group A - 26.2 Group B - 25.9	F:144 M: 2	NR	Y	NR	NR	NR	2-0 Vycril	Interrupted
54	Safety and Efficacy of Outpatient Drainless Abdominoplasty: A Single-Surgeon	Alisha B Paranzino, Jason Sims, David S Kirn; 2023	Retrospective study	D:194 PTS:260	454	0.806	PTS: 43.7 D: 40.2	D: 25.5 PTS: 26	M:11 F:443	10-15 interrupted sutures from subxyphoid to caudally	Y	NR	NR	NR	Vycril 1	Interrupted
55	Safety of Fleur-de-lis Abdominoplasty after Massive Weight Loss.	Joshua J DeSerres, Tanis Quaife, Alexander Morzycki, Matthew W T														
56	Scarpa Fascia Preservation to Reduce Seroma Rate on Massive Weight Loss	Oskari Repo, Carlo M. Oranges, Pietro G. di Summa, Panu Uusalo, Mikael Anttinen, and	Retrospective study	D	202	NR	44	28	F:173 M:29	N	NR	NR	NR	NR	N	N
57	Seroma formation following abdominoplasty: a retrospective clinical review	U. D. Khan/2011	Retrospective	Group A(53)- Abdominoplasty with umbilical	121	1	Group A: 37 ± 6.7	Group A: 25.9 ± 4.1	NR	NR	NR	NR	NR	NR	2-0 Vycril	Interrupted
58			Clinical Review	relocation and also had liposuction			Group B: 39.5 ± 9.5	Group B: 26.2 ± 4.1								
59				of hips and flanks with no PTS			Group C: 40.6 ± 10.9	Group C: 27.6 ± 4.9								
60				Group B(24) - Abdominoplasty with umbilical												
61				relocation and also had liposuction												
62				of hips and flanks with PTS												
63				Group C(44) - Abdominoplasty with limited												
64				supra-umbilical undermining,												
65				umbilical also had liposuction												
66				of hips and flanks												
67	Seroma Rates Are Not Increased When Combining Liposuction With Progressive	Daniel J Gould, MD, PhD, Luis H Macias, MD, Fouad Saeg, BS, Phillip Dauwe, MD,	Retrospective	299 patients with	619	0.8	Mean age: 45	BMI: 25.5	F:598 M:22	Y not specified	NR	NR	NR	NR	NR	NR
68			cohort study	drainless PTS												
69				320 patients with												
70				drains only												
71	The Effect of Drains and Compressive Garments Versus Progressive	Tim Brown, Kayla Murphy, Tracey Murphy/2019	Retrospective cohort study	D: 61 PTS:171	232	NR	42.1	NR	M:3 F:299	Y not specified	Y	N	N	N	1/0 Vicryl	Interrupted
72																
73	The Effect of Drains and Compressive Garments Versus Progressive															
74	The Impact of Perioperative Blood Pressure on Hematoma Development After	Megan Rudolph, Laura Galazra, Madison Greer, Kshipra Hemal, Christopher	Retrspective cohort study	D	143	1	50.8	37.9	F:123 M:20	N	NR	NR	NR	NR	NR	NR
75	The Internal Negative-Pressure Wound Control System: A Paradigm Shift for															
76	The no-drain, no-quilt abdominoplasty: a single-surgeon series of 271	Quaba, F.R.C.S.(Plast.). Conlin. 2014	Retrospective chart analysis	No PTS & Liposuction	271	0.61	44	26.6	F:266 M:5	N	Y	Y	N	N	N	N
77	The timing of drain removal-empiricism or science?.	Mohammed Sultan Ul Hassan &	Prospective study	D	22	NR	35	BMI ≤30	F:22	N	Y	N	N	N	N	N
78		Justin Sanjay Chatterjee & Zeiad Alshameeri / 2011														
79	The Use of Quilting Suture in Abdominoplasty Does Not Require Aspiratory	H. L. Arantes, R. G. Rosique, M. J. F. Rosique and J. M. Melega 2009	Retrospective study	PTS:28 D+PTS:32	60	NR	NR	NR	F:60	Bilateral to the median line	NR	NR	NR	NR	Vicryl 2-0	Interrupted
80	Use of a Lysine-Derived Urethane Surgical Adhesive as an Alternative to	Michelle A. Spring, 2018	Prospective study	PTS 10 and ADHESIIVE WOUND CLOSURE 10	20	1	45.05	26.1	F:20	anchoring	NR	NR	NR	Y	2-0 Polysorb	Interrupted

	Scarpa (Y= preserve	Drainage technique	Drain total output	Drain removal	Lipoabdominoplasty	Seroma (n)	SEROMA %	Method of seroma ca	Haematoma (n)	Haematoma %	Flap Necrosis (n)	Flap necrosis %	Re-operation (n)	Re-operation (%)	Followup
53	NR	vacuum drains	Group A: 197cc. Group B: 111cc	3 days	Standard adominoplasty	Group A: 25 Group B: 2	Group A: 26% Group B: 4%	Clinical	Group A: 0 Group B: 0	Group A: 0% Group B: 0%	NR	NR	NR	NR	6 months
54	N	vacuum drains	NR	NR	Modified, adominoplasty, full adominoplasty, Belt lipectomy	D:48 PTS:0	D:24.7% PTS:0	Clinical	NR	NR	NR	NR	D:1 PTS:0	D:0.5% PTS:0%	9 months
55															
56	Y:53 N:149	vacuum drains	277,5	<30ml/day	Standard adominoplasty, Scarpa fascia preservation adominoplasty	44	0.21	Clinical /Ultrasound	25	0.123	10	0.0495	NR	NR	46.5 months
57	N	Mean in first 24 hrs:	A:134 ; B:62 ; C:75	NR	Lipoadominoplasty	Group A: 14	Group A: 25.5% Group B: 7.7% Group C: 0%	NR	A: 14 B:2 C:0	A: 25,5% B:7.7% C:0	NR	NR	NR	NR	6
58		Group A: 134 ml (20-574)				Group B: 2									
59		Group B: 62 ml (0-129)				Group C: 0									
60		Group C: 75 ml (0-280)													
61															
62															
63															
64															
65															
66															
67	NR	vacuum drains	NR	NR	Standard adominoplasty, lipoadominoplasty	PTS:8 D:27	PTS: 2.6% D:8.5%	Clinical	NR	NR	NR	NR	NR	NR	9.3 months
68															
69															
70															
71	N	vacuum drains	NR	3 days	modified	PTS: 10 D:10	D:16.4% PTS:6.2	Clinical	NR	NR	NR	NR	NR	NR	12 weeks
72					Pitanguy technique										
73															
74	NR	vacuum drains	NR	NR	Low transverse incision /fleur-de-lis	NR	NR	NR	7	0.05	NR	NR	5	0.071	3.2 months
75															
76	N	N	N	N	full adominoplasty	21	0.077	Clinical	5	0.018	1	0.003	7	0.026	6.2 months
77	NR	vacuum drains	N	A:<30ml/day B:<50ml/day	NR	5	0.227	NR	1	0.045	0	0	NR	NR	NR
78															
79	N	vacuum drains	31.4 ml (mean per day)	<30ml/day	standard adominoplasty	PTS: 1 PTS + D: 1	PTS: 3.5% PTS + D: 3.12%	Clinical	NR	NR	NR	NR	NR	NR	6 months
80	NR	N	N	N	Standard adominoplasty	0	0	Clinical	0	0	NR	NR	NR	NR	NR

81										the flap fascia to abdominal wall fascia																					
82	Use of Quilting Sutures During Abdominoplasty to Prevent Seroma Formation: Are	M. Sforza; R. Husein; K. Andjelkov; P. C. Rozental-Fernandes; R. Zacchedu;	Retrospective study	Group A (100)- No PTS & x2 Drains	414	1	Group A: 41.2	25-35	F: 414	9 to 15 single sutures	NR	NR	NR	NR	Monocryl 2.0	Interrupted	N	vacuum drains	NR	<30ml/day / 48 hours	lipoabdominoplasty	Group A: 12	Group A: 12%	Clinical	Group A: 2	Group A: 12%	NR	NR	Group A: 0	Group A: 0	1 year
83				Group B (226) - PTS & x2 drains			Group B: 40.2															Group B: 0	Group B: 0%		Group B: 0	Group B: 0%			Group B: 0	Group B: 0	
84				Group C (88)- PTS & 1 drain			Group C: 41.5															Goup C: 0	Group C: 0%		Goup C: 0	Group C: 0%			Goup C: 0	Goup C: 0	
85	Use of the PlasmaJet system in patients undergoing abdominal	A. Iannelli; A. S. Schneck; J. Gugenheim/2010	Prospective Randomized Clinical trial	D	60	0	41.7	BMI <35	F: 59 M: 1	N	Y (29 patients)	N	Y (PJS)(31 patients)	NR	N	N	N	vacuum drains	330ml	<30ml/day	standard adominoplasty	1	0.0345	Clinical	2	0.0333	NR	NR	NR	NR	3 months
86	What Is the Impact of Comorbidities on the Risk for Postoperative Body-Contouring	S. C. Rosa; J. L.S. de Macedo; L.R. Canedo; L.A. Casulari/2018	Prospective study		139		41.18	27.44	F: 130 M: 9																						

Supplementary Table 3A Characteristics of the included studies. Single arm studies have also been chronicled for completeness[9,26-45]. 3B Broader characteristics of studies including additional detail for reference.
