# Supplementary material for: A Systematic Review and Meta-Analysis Evaluating the Surgical Outcomes of Progressive Tension Suturing Compared to Drains in Abdominoplasty Surgery
Source: Aesthet Surg J. 2024 Jul 30;45(1):71–83. doi: 10.1093/asj/sjae171 (PMC11634385; doi:10.1093/asj/sjae171)
Supplement: sjae171_Supplementary_Data [file sjae171_supplementary_data.zip › Supplementary Table 4.pdf]

Author / Year	*BMI (compare outcomes between normal BMI < 25 v/s overweight BMI 25-30 v/s over 30)*	*Gender (Female:Male)*	*Technique (quilting, Scarpa's preservation, etc)*	*Was a thin layer of fatty tissue preserved over fascia?*	*Type and technique of quilting suture (e.g., 2-0 vicryl)*	*How many sutures and where were they placed?*	*Surgical time*	*Method of seroma detection (fluctuant swelling on clinical exam v/s US)*	*Follow-up duration*	*Total drain output and time to drain removal*	*Technical manoeuvres in each study (electrocautery, scalpel, ultrasonic scalpel)*	*Tissue adhesives/glue*
N. Agochukwu-Nwubah et al. (2021)	Dependent on operation but average: 26.5	340:11	Scarpa preserved	NR	2-0 polydioxanone suture	15-20	Total not reported, provided how much additional time placing PTS caused (15-20 mins)	Clinically diagnosed; 1 case of a dry aspiration (criteria for seroma include anything >1 mL)	1 year	No drains used	NR	NR
P. Andrades et al (2007)	Group 1: 26.7 ± 2.5<br>Group 2: 27 ± 2.1<br>Group 3: 25.3 ± 5.3<br>Group 4: 26.4 ± 2.5	100:0	Midline fascia plication was performed in one layer	NR	Running, non-absorbable suture. fixation and advancement sutures also used. PTS too	30-40	Surgical time was longer by 50 mins in groups 2 and 4	Ultrasound	6 months	7 days	Electrocautery	NR
J. W. Antonetti et al (2010)	NR	508:9	Superior abdominal flap elevation, lateral dissection was never extended to the costal margin. preserves epigastric arteries	NR	2-0 vicyrl sutures	NR	NR	NR	NR	NR	Scalpel	NR
H. L. Arantes et al (2010)	NR	50:0	Quilting suture + aspiratory drainage	NR	2-0 vicyrl sutures	NR	NR	NR	6 months	Group 1: No drains<br>Group 2: average output 31.4ml in last 24hrs. Drain was retained on average for 5 days	Scalpel	Adhesion sutures used
A. Arnaout et al (2020)	NR	NR	NR	NR	Bilateral barbed PTS 3-0 V-LocTM	NR	NR	NR	NR	NR	NR	NR
R. A. Baxter (2001)	NR	NR	Tension on the flap is	No	Non absorbable braided sutures.	Placed in rectus fascia	NR	Percutaneous fluid seen underflap	1-42 months	NR	Electrocautery, "dog ear"	NR

			distributed by suturing the superficial fascia on the underside of the flap to the deep fascia		OO polydioxanone. anchor suture			regarded as seroma, via clinical examination			technique for excision	
M. Bromley et al (2018)	BMI mean; Group 1: 27.8 Group 2: 27.5 Group 3: 26.9 Seroma incidence; Group 1: 16 Group 2: 3 Group 3: 2	49:14	Scarpa preserved, PTS quilting sutures	No	2-0 Mononylon X-type suture, 3-0 Vicryl, Nylon 3-0, 4-0, Monocryl 3-0		Group 2 (mean): 29.81 Group 3 (mean): 13.38	Clinical diagnosis and ultrasound	90 days	Days of drain removal; group 1: 4.57 group 2: 1.95 group 3: 2.19	Electrocautery	NR
J. V. Cucchiaro et al (2017)	NR	272:4	Scarpa's fascia preserved	Yes	2-0 Mononylon X-type suture, 3 Vicryl 2-0 running suture	Scarpa's fascia	PTS added 3-5 mins of time to total surgical time	Ultrasound	Minimum 2 years	NR	Monopolar double clamp	NR
S. Gallagher et al (2018)	Group A: 26.6 Group B 24.6 Complications correlated to BMI $p = 0.88$	NR	The sutures secured the superficial (Scarpa) fascia to the deep abdominal fascia.	NR	0 Vicryl sutures	An average of 25 to 30 progressive tension sutures were placed per case, Scarpa's fascia	Group A: 313 mins Group B: 228 mins	Clinically detected	Maximum 3 years	NR	NR	NR
S. Khan et al (2006)	PTS group: 26.4 Non-PTS group: 25.7 Complication in PTS group: 14.3% Complication in non-PTS group: 44.4%	NR	NR	NR	0 Vicryl suture	NR	PTS group mean: 106 mins non-PTS group mean: 127	Clinically diagnosed	PTS mean: 5.42±3.64 non-PTS group mean: 13.41±15.1	Drain removal PTS group: 9.2±4.3 non-PTS group: 11.9±7.9	Electrocautery	NR
U. D. Khan et al (2011)	Group A BMI: 25.9±4.1 Group B BMI: 26.2±4.1 Group C BMI: 27.6±4.9 Group A seroma incidence: 25.5% Group B seroma incidence: 7.7% Group C seroma incidence: 0%	NR	PTS in groups B and C	NR	2-0 Vicryl suture	NR	NR	Clinically diagnosed	NR	NR	Scalpel	NR
L. H. Maclas et al and W. Grant Stevens/ (2016)	Drains group BMI: 25.4 PTS group BMI: 26 Seromas D-	433:18	PTS quilting technique	Yes	0-vicryl quilting sutures	10-12 sutures total in the supra/infraumbilical sagittal plane region	NR	NR	Mean: 6 months range: 1-32 months	NR	Electrocautery, sutures closed in layered fashion	NR

	group: 9%<br>PTS group: 2%											
A. T. Mohan, et al. (2015)	b-PTS BMI mean: 28.2<br>non-PTS drain BMI mean: 29.6<br>donor site complication b-PTS: 21.4%<br>donor site complications non-PTS: 35.3%	NR	Barbed-PTS quilting, Scarpa's fascia preserved	NR	2/0 Quill PDO barbed suture, 2-0 Vicryl suture	Supraumbilical	B-PTS group mean: 557 min<br>standard close group mean: 626 min	NR	Length of mean hospital stay:<br>b-PTS: 3.9<br>non-PTS: 4.7	NR	Scalpel	NR
D. Parvizi et al. (2015)	Mean BMI: 29<br>seromas: 5%<br>hematomas: 7%	182:23	NR	NR	NR	NR	NR	Clinical diagnosis	Mean: 5.94 years<br>range: 4-9 years	Up to 25 days for drain removal	NR	NR
J. F. Pascal et al (2008)	NR	NR	Quilting sutures, keeping lymphatics associated with Scarpa's fascia intact	NR	NR	NR	NR	NR	NR	NR	NR	NR
Pollock et al (2012)	Mean BMI: 29.1<br>local complications: 4.2%	587:10	NR	No	0-0 PTS barbed suture	3 sutures, near mons	NR	NR	Mean: 13.7 months	NR	Electrocautery, scalpel	NR
A, Rosen et al (2019)	NR (no data on BMI)	431:14	No Scarpa preservation	NR	All barbed PTS suture - #2 polypropylene Quill, followed by a layer of #2 PDO Quill.	Rectus fascia	NR	Physical examination	(Up to) 6 months	NR	NR	No
Marcos Sforza, et al (2015)	Group 1/2/3: same BMI range 25-35 so can't differentiate outcomes based off of BMI	539:0	Quilting sutures	NR	Quilting sutures	9-15 depending on size of the patient. abdominal wall fascia	1.5 to 2 hours	Clinical examination	1 year	NR	Avelar's technique	NR
Patricio Andrades, M.D. et al (2007)	BMI was reported to not have affected outcomes	60:0	PTS quilting sutures	NR	Fixation and advancement sutures	30-40	Operating time longer by 50 mins in groups 2 and 4	Ultrasound, clinical exam	6 months	200ml+. 7 days	Electrocautery	NR

Supplementary Table 4 Additional information pertaining to the included studies[46] including operative details and patient group parameters. Single arm studies have also been chronicled for completeness[9,26-45]
